# Draft genome sequence of *Limosilactobacillus fermentum* PJG11 isolated from wild yam, *Dioscorea luzonensis* in Ilocos Norte, Philippines

**DOI:** 10.1128/mra.00325-26

**Published:** 2026-04-23

**Authors:** Ma. Joy Theresa Agcaoili, Lemuel Ray Balloyan, Jimmbeth Zenila Fabia, Marvielyn Olivar, Patrick James Macugay, Peter James Icalia-Gann

**Affiliations:** 1 Center for Cellular and Molecular Medical Research, College of Medicine, Mariano Marcos State University 69374, Batac, Ilocos Norte, Philippines; 2 Research Directorate, Mariano Marcos State University 69374, Batac, Ilocos Norte, Philippines; 3 Department of Biological Sciences, College of Arts and Sciences, Mariano Marcos State University 69374, Batac, Ilocos Norte, Philippines; 4 Department of Biochemistry and Molecular Biochemistry, College of Medicine, Mariano Marcos State University 69374, Batac, Ilocos Norte, Philippines; Nanchang University, Nanchang, Jiangxi, China

**Keywords:** whole genome sequencing, probiotics

## Abstract

This report presents the draft genome sequence of a lactic acid bacterium, *Limosilactobacillus fermentum* PJG11, isolated from wild yam, *Dioscorea luzonensis*, an endemic root crop in Ilocos Norte, Philippines. The discovered genomic characteristics of the strain shall have implications in the study of its beneficial properties and toward comparative genomic analyses involving strains of the same species.

## ANNOUNCEMENT

Lactic acid bacteria (LAB) have been established as key sources of probiotic strains exhibiting genomic and metabolic characteristics that offer health benefits toward their hosts (1–3), with the potential to treat certain cancers as one of them (4, 5). Studies have highlighted *Limosilactobacillus fermentum* strains as one of many probiotic candidates demonstrating significant anticancer treatment properties *in vitro* and *in vivo* (6–10). Here, the genome of a locally isolated *L. fermentum* strain designated as PJG11 was sequenced and assembled hoping to contribute an in-depth understanding and aid in the investigation of potential locally-isolated anticancer probiotic treatments.

Strain PJG11 was isolated from the indigenous *Dioscorea luzonensis* harvested in Ilocos Norte, Philippines (18.0564° N, 120.5650° E). Plant tubers were initially peeled, cut into smaller cubes, and then washed with sterile distilled water. The cut tissues were then submerged in de Man, Rogosa, and Sharpe (MRS) broth and incubated for 4 days. The resulting solution was then directly streaked into MRS agar and incubated at 37°C for 24–48 h. Colonies displaying typical LAB morphology were picked and subjected to successive re-streaking with the same medium and growth conditions. Pure cultures were preserved with 50% (vol/vol) glycerol and stored at −80°C for long-term storage.

For whole genome sequencing, the PJG11 isolate was revived and cultured in 20 mL of MRS broth and incubated with shaking for 24 h at 37°C. Cells were collected through centrifugation of approximately 1.5 mL of broth culture for DNA extraction using the GF-1 Bacterial DNA Extraction Kit (Vivantis Technologies, Malaysia) following the manufacturer’s protocol. Quality and concentration of the extracted DNA was assessed by agarose gel electrophoresis and ScanDrop2 spectrophotometer (Analytik Jena, Germany). DNA extracts were submitted to the DNA Sequencing Core Facility of the Philippine Genome Center for library preparation and sequencing. DNA libraries were constructed using the Nextera XT DNA Library Preparation Kit (Illumina Inc., USA) and assessed using the Agilent TapeStation 2200 system (Agilent Technologies, USA). The whole genome was sequenced using the Illumina MiSeq platform (Illumina Inc., USA) with a paired-end read of 150 bp.

Read qualities of raw sequencing data were assessed using FastQC v0.12.0 (11), in which read pairs totaled 4,241,473, with the number of bases amounting to 638.4 Mbp. Reads with a Phred score below Q20 and <36 bp in length were trimmed using Trimmomatic v0.40 (12). *De novo* genome assembly was conducted using SPAdes v4.2.0 (13). The quality of the refined assembly was assessed using QUAST v5.3.0, CheckM2 v1.1.0, and BUSCO v6.0.0 using the lactobacillaceae_odb12 parameter (14–16). The genome was annotated using Bakta v.1.11.3 (17) in compliant mode. Default parameters of all software were applied unless stated otherwise. Taxonomic identification and ANI calculation using the MiGA webserver reveal the isolate’s closest relative as *Limosilactobacillus fermentum* LMG5902 with an ANI of 97.86% (18). Assembly and annotation are summarized in Table 1.

**TABLE 1:** Summary of the assembly and annotation results of *L. fermentum* PJG11

Parameter	Value
No. of Contigs	173
Genome Size	2,028,528 bp
GC Content	51.68%
Ave. Coverage Depth	618x
N50	40,877
L50	14
Coding Density	85.4%
CheckM2 Completeness	99.98%
Contamination	0.11%
BUSCO Completeness (lactobacillaceae_odb12)	98.8%
Protein-coding Genes	1964
tRNA	59
ncRNA	5
ncRNA-regions	25

## Data Availability

The BioProject accession in which the genome data is a part of is PRJNA1293312. Additionally, raw reads can be accessed with the SRA accession SRR35991899, whole genome shotgun data via the GenBank WGS master accession JBSNBV000000000, and bacterial sample information with the BioSample accession SAMN53118818.
